# Dendrobine alleviates CCl_4_-induced acute liver injury by inhibiting necroptosis through activation of the Nrf2/PPARγ/SOD2 signaling pathways

**DOI:** 10.1186/s13020-025-01241-8

**Published:** 2025-11-02

**Authors:** Yonggang Yang, Xiaolong Fu, Ling Tan, Siting Xian, Naiyu Fan, Qinglin Gan, Nan Nan, Lizhen Hu, Jingshan Shi, Qin Wu, Shaoyu Zhou

**Affiliations:** 1 Key Laboratory of Basic Pharmacology of Ministry of Education and Joint, International Research Laboratory of Ethnomedicine of Ministry of Education, Zunyi Medical University, Zunyi, 563100 Guizhou China; 2 School of Pharmacy, Zunyi Medical University, Zunyi, 563100 China

**Keywords:** Acute liver injury, Dendrobine, PPARγ, Nrf2, MLKL

## Abstract

**Background:**

Acute liver injury (ALI) represents a critical clinical challenge characterized by rapid degradation of hepatic function, necessitating prompt intervention for improved patient outcomes. Dendrobine (DDB) is the main bioactive component of *Dendrobium nobile* Lindl. (DNL), a traditional Chinese herb renowned for its protective effects against liver injury. However, the specific mechanisms underlying its hepatoprotective effects have not yet been fully elucidated.

**Objective:**

This study aims to elucidate the potential mechanism underlying the protective effects of DDB against ALI, particularly through the Nrf2/PPARγ/SOD2 pathways, to provide a scientific basis for its application in ALI treatment.

**Methods:**

CCl_4_-induced ALI models in animals were used to evaluate DDB's therapeutic effects via biochemical and pathological analyses. The regulation of the Nrf2/PPARγ/SOD2 axis and mitochondrial ROS (mtROS) by DDB was observed through WB, RT-qPCR, and Mito-SOX assays, which was confirmed both *in vitro* and *in vivo*. JASPAR predictions and ChIP assays validated PPARγ's regulation of SOD2. DDB’s interaction with Keap1 was assessed by molecular docking, cellular thermal shift assay (CETSA), and drug affinity responsive target stability (DARTS).

**Results:**

DDB effectively suppressed MLKL activation and significantly alleviated ALI. DDB also upregulated Nrf2/PPARγ/SOD2 expression and reduced mtROS production. Further studies using pharmacological approaches showed that PPARγ activation increased SOD2 expression, reduced p-MLKL, and lowered mtROS levels. Conversely, PPARγ inhibition reversed these effects and diminished DDB's efficacy. Silencing Nrf2 *in vivo* decreased PPARγ/SOD2 expression and activated MLKL, counteracting DDB's protective effects. Overexpression of Nrf2 prevented the decrease in PPARγ/SOD2 protein expression induced by CCl_4_, and inhibited mtROS release and MLKL activation, indicating that Nrf2 regulates p-MLKL via the PPARγ/SOD2/mtROS axis to suppress necroptosis. Analysis using JASPAR revealed that the SOD2 promoter contains a PPARγ response element. ChIP assays showed that Nrf2 activated SOD2 through PPARγ, not directly transcriptionally regulated SOD2. Further studies demonstrated that DDB interacted with Keap1 to promote Nrf2 nuclear translocation, thereby protecting the liver from damage.

**Conclusion:**

This study demonstrates that DDB inhibits mtROS and p-MLKL through the Nrf2/PPARγ/SOD2 signaling axis, thereby suppressing necroptosis and ameliorating ALI.

**Graphical Abstract:**

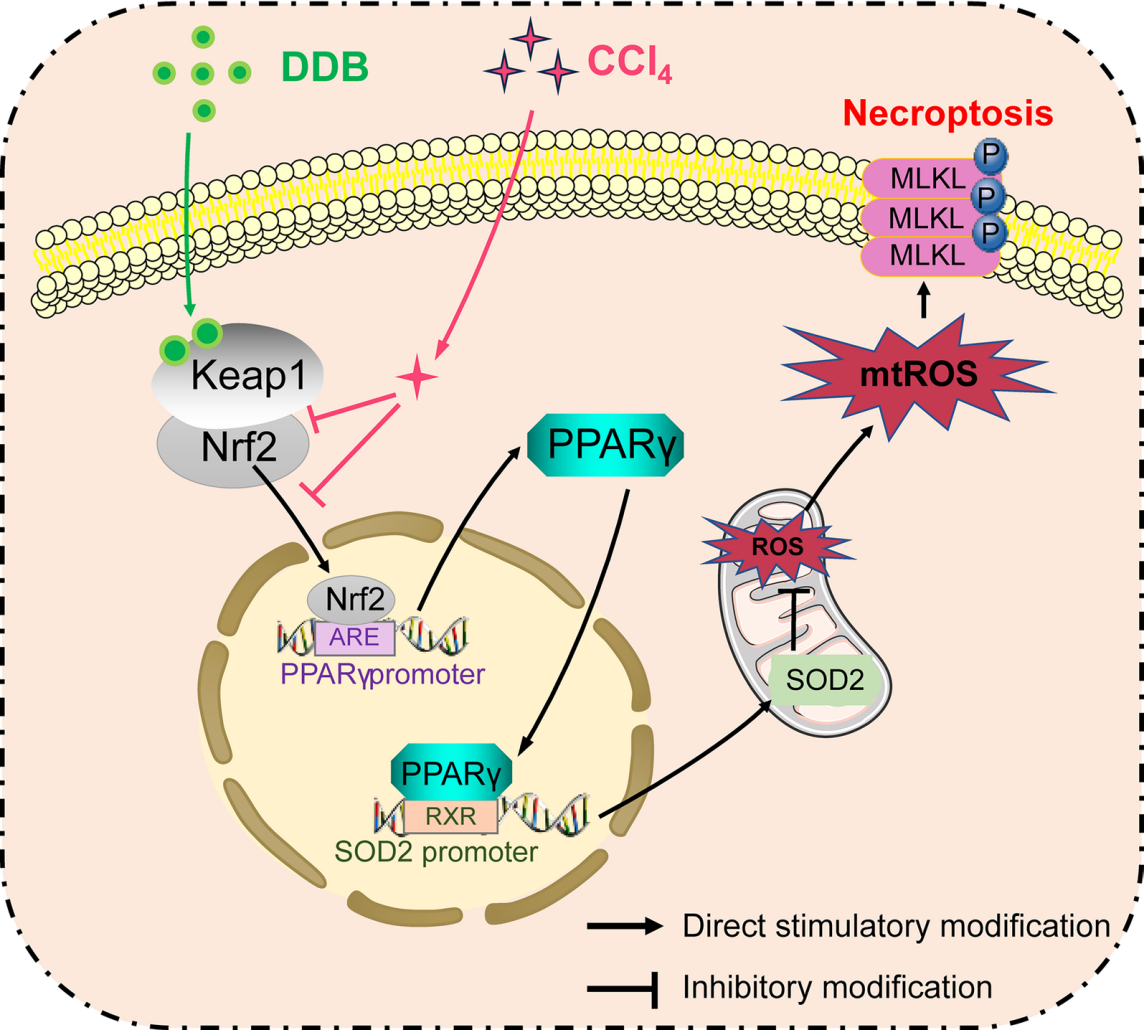

**Supplementary Information:**

The online version contains supplementary material available at 10.1186/s13020-025-01241-8.

## Introduction

The liver is the primary detoxification organ, during which process it is also vulnerable to assault from foreign substances such as chemicals, viruses, various medications, and bacterial metabolites, leading to hepatocyte necrosis or apoptosis and subsequent liver injury [1]. Over the past few decades, there has been a marked increase in the prevalence of liver disease, which has become one of the most significant causes of morbidity and mortality on a global scale [2]. Therefore, there is an urgent need to develop more effective drugs for the prevention and treatment of liver disease.

Necroptosis is a form of programmed necrosis mediated by RIPK1, RIPK3 and MLKL [3]. MLKL is a critical regulatory protein in the necroptosis pathway, which has been implicated in pathological mechanisms of various diseases, and the necroptosis process absolutely depends on the activation of MLKL [4]. Recent studies have emphasized the critical role of necroptosis in liver diseases [5]. Notably, a survey of patients with hepatitis B virus-related liver failure revealed that the protein expression levels of RIPK3 and MLKL in patient liver tissues were significantly higher than those in healthy individuals [6]. Furthermore, MLKL knockout (MLKL^−/−^) in mice significantly ameliorated clinical symptoms of liver injury and fibrosis induced by CCl_4_ and bile duct ligation [7]. These studies indicate a critical role of regulation of necroptosis in the mechanism of liver injury. Studies have reported that oxalate can damage mitochondria and ultimately activate MLKL, triggering necroptosis and leading to acute kidney injury [8]. Furthermore, our studies have shown that inhibiting the activation of necroptosis by regulating mitochondrial ROS (mtROS) can protect against acute liver injury (ALI) [9]. However, the regulatory mechanism of MLKL activation in the context of ALI has yet to be fully elucidated.

Nrf2 is an antioxidant transcription factor that counteracts oxidative stress by upregulating the expression of a series of antioxidant enzymes, including HO-1 and SOD2 [10]. Under normal conditions, Nrf2 is bound to Keap1 and sequestered in the cytoplasm. Upon external stimulation, Nrf2 dissociates from Keap1, translocates to the nucleus, binds to the antioxidant response element (ARE) in the promoter regions of target genes, and activates the transcription of downstream molecules [11]. PPARγ is a ligand-activated transcription factor that can suppress the generation of superoxide anion radicals and induce the expression of antioxidant enzymes [12]. Studies have shown that Nrf2 induces PPARγ expression by binding to the ARE in the PPARγ gene promoter, thereby alleviating oxidative inflammation [13]. The activation of both Nrf2 and PPARγ can ameliorate liver injury, as they play pivotal roles in liver disease progression by regulating the expression of genes involved in critical processes such as hepatic metabolism, energy homeostasis, and oxidative stress [14–16]. Among these, mtROS is recognized as one of the factors they regulate [17, 18]. Our research has confirmed that mtROS can activate the necroptosis pathway, leading to hepatocyte injury, whereas overexpression of SOD2 effectively inhibits this process [9]. Currently, many studies have shown that Nrf2, PPARγ, and SOD2 can all regulate mtROS [19–21]. However, the relationship among these three molecules, especially the role of these proteins in necroptosis in liver injury, remains far from elucidated. Dissecting the interaction of these proteins and its role in the regulation of necroptosis in liver injury holds significant scientific importance for the development of new therapeutic approaches and strategies.

*Dendrobium nobile* Lindl. (DNL) has been widely used in traditional Chinese ethnomedicine for thousands of years and is included in the Pharmacopoeia of China (2020 Edition). Studies have shown that DNL plays an important role in the prevention and treatment of liver diseases, with its underlying mechanisms involving the activation of the Nrf2 signaling pathway to alleviate oxidative stress in the liver [22–24]. Recently, we found that its protective effects are also associated with the inhibition of necroptosis through the regulation of mitochondrial-derived mtROS [9]. Emerging research has increasingly focused on dendrobine (DDB), a major bioactive compound extracted from DNL. Studies have shown that DDB can reduce oxidative stress injury via the Nrf2 pathway [25]. Additionally, DDB has been reported to decrease intracellular ROS production, thereby ameliorating cellular senescence, osteoarthritis, and lung injury [26, 27]. However, reports on the therapeutic effects of DDB in liver injury are currently limited. Existing studies indicate that DDB alleviates liver injury by modulating inflammation, immune responses, and PPAR-mediated lipid metabolism [28, 29]. Nevertheless, it remains unclear whether DDB exerts its hepatoprotective effects by activating the Nrf2/PPARγ/SOD2 signaling axis to suppress necroptosis.

This study elucidates that in the context of liver injury, DDB activates Nrf2, which subsequently transcriptionally upregulates PPARγ to enhance SOD2 expression, rather than directly targeting SOD2. DDB inhibits mtROS and p-MLKL through the Nrf2/PPARγ/SOD2 signaling axis, thereby suppressing necroptosis and ameliorating ALI.

## Materials and methods

### Animals

All experiments were conducted using 6–8 week-old male C57BL/6J mice weighing between 18–22 g. Wild-type (WT) mice were purchased from Hunan SJA Laboratory Animal Co., Ltd. (Hunan, China). Nrf2 knockout (Nrf2^−/−^) mice were a kind gift of Dr. Lili Ji of the Institute of Chinese Medicine, Shanghai University of Traditional Chinese Medicine. MLKL^−/−^ mice were generously provided by professor Zhiyong Zhang of the State University of New Jersey, New Brunswick, NJ, USA. All animals were maintained in an environmentally controlled room with a 12 h light/dark cycle at a controlled temperature (18–25 ℃) and were acclimatized for at least 7 days with free access to food and water before the experiments. This study was conducted according to the NIH Guide for the Care and Use of Laboratory Animals and approved by the Institutional Animal Use and Care Committee of Zunyi Medical University (Approval No. SCXK 2012-0005).

### Chemicals

Dendrobine (DDB) (99% purity) was purchased from Beijing Meirenda Technology Co. Other chemicals and reagents employed in this study included colza oil (Huida Grain and Oil Co., Mianyang, China), CCl_4_ (Kelong Chemicals Co., Chengdu, China), pioglitazone (HY-13956, MCE, China), GW9662 (HY-16578, MCE, China), and ML385 (HY-100523, MCE, China).

### Maintenance of AML-12 cell line

The AML-12 hepatocyte cell line was obtained from Procell Life Science & Technology Co., Ltd. The AML-12 cells were cultured in DMEM supplemented with 10% fetal bovine serum and 1% penicillin/streptomycin (P/S) at 37 ℃ in a humidified atmosphere containing 5% CO₂. Acute cell injury model was established by stimulating the AML-12 cells with 20 mM CCl_4_ for 24 h.

### Experimental design

WT mice were randomly assigned to seven groups: Control, DDB, GW9662, CCl_4_ (20 μL/kg), DDB (20 mg/kg) + CCl_4_, GW9662 (1 mg/kg) + CCl_4_, and DDB + GW9662 + CCl_4_. DDB was dissolved in PBS containing 1% Tween 80. Animals were administered drugs once daily via oral gavage according to body weight. The DDB group, DDB (20 mg/kg) + CCl_4_ group, and DDB + GW9662 + CCl_4_ group received DDB by gavage for 7 consecutive days, while the remaining groups received PBS containing 1% Tween 80 at the same time points. On day 5, the GW9662 group, GW9662 (1 mg/kg) + CCl_4_ group, and DDB + GW9662 + CCl_4_ group were administered GW9662 by gavage 1 h after receiving PBS containing 1% Tween 80 or DDB, while the other four groups received the vehicle. This dosing regimen was continued until day 7. On day 7, CCl_4_ (dissolved in vegetable oil) was administered intraperitoneally to the CCl_4_ (20 μL/kg) group, the DDB (20 mg/kg) + CCl_4_ group, the GW9662 (1 mg/kg) + CCl_4_ group, and the DDB + GW9662 + CCl_4_ group 1 h after the final gavage of GW9662 to induce liver injury, while the remaining groups received vegetable oil.

MLKL^−/−^ mice were divided into two groups: Control and CCl_4_ (20 μL/kg). The control group received vegetable oil, while the model group received CCl_4_. Nrf2^−/−^ mice were divided into three groups: Control, CCl_4_ (20 μL/kg), and DDB (20 mg/kg) + CCl_4_. For the DDB (20 mg/kg) + CCl_4_ group, DDB was administered orally once daily for seven consecutive days. The control group and the CCl_4_ (20 μL/kg) group received PBS containing 1% Tween 80 by gavage at the same time points for 7 days. One hour after the final DDB administration, the CCl_4_ (20 μL/kg) group and the DDB (20 mg/kg) + CCl_4_ group were injected intraperitoneally with CCl_4_, while the control group received vegetable oil. Mice were fasted for 12 h after CCl_4_ injection but allowed free access to water. Twenty-four hours after injection, mice were euthanized, and blood and liver tissues were collected. A portion of the liver tissue was fixed in 10% neutral buffered formalin for histological analysis, and the remaining tissue was stored at – 80 ℃ for subsequent experiments.

### Biochemical assays of ALT and AST

Blood was obtained from the orbital vein of anesthetized mice for subsequent serum biochemistry analysis. The blood samples were stored at room temperature (RT) for 2 h, then centrifuged for 10 min at 3000 rpm at 4 ℃ to separate the serum, which was kept at – 80 ℃ until use. The alanine aminotransferase (ALT, catalog #C009-2) and aspartate aminotransferase (AST, catalog #C010-2) levels were measured strictly in accordance with the instructions provided by the commercial biochemical kits (Jiancheng, Nanjing, China).

### Pathologic histology evaluation

Liver tissues were collected from experimental animals and immediately fixed in 10% neutral formalin at RT for 48 h. After fixation, the tissues were processed for paraffin embedding. Samples were sectioned at 4 μm thickness using an RM2235 microtome (Leica, Germany) and stained with hematoxylin and eosin (H&E). Finally, the slides were coverslipped using a mounting medium. The stained sections were examined under a light microscope (Olympus, Japan) at various magnifications (10 ×, 40 ×) to assess histological changes. Representative images were captured using a digital camera attached to the microscope.

### Western blot assay

Samples were processed following the general steps for Western blotting (WB). Membranes were incubated overnight with primary antibodies against RIPK1 (1:1000, #3493, CST), p-RIPK1(1:1000, #53286, CST), p-RIPK3(1:1000, #91702, CST), RIPK3 (1:1000, #10188, CST), MLKL (1:1000, #37705, CST), p-MLKL (1:1000, #37333, CST), SOD2(1:5000, #24127-1-AP, Proteintech Group), PPARγ (1:5000, #66936-1-lg, Proteintech Group), Nrf2 (1:1000, #166396-1-AP, Proteintech Group), Keap1 (1:2000, ab227828, Abcam), Lamin B (1:5000, #12987-1-AP, Proteintech Group) and GAPDH (1:5000, #ET1601-4, HUABIO). This was followed by a 1 h incubation with horseradish peroxidase-conjugated secondary antibodies (1:5000) at RT. Immunoblots were visualized using a chemiluminescence reagent BeyoECL Plus (Beyotime, Shanghai, China), gray values of each blot were analyzed using Image J software (Bio-Rad, Hercules, California, USA), and the expression levels of target proteins were represented as the ratio of the gray value of the target protein to that of GAPDH.

### Quantitative real-time PCR (RT-qPCR)

Total RNA was extracted from liver tissue or AML-12 cells using RNA iso Plus (Takara Bio Inc., Japan). The quantity and quality of RNA were determined spectroscopically using an ultra-micro spectrophotometer. Total RNA was used to synthesize cDNA using the M-MLV reverse transcriptase kit (Thermo Fisher) according to the manufacturer’s protocol. The cDNA samples were used for RT-qPCR in a total volume of 15 µl using SYBR Green Reagent (Roche Diagnostics, Meylan, France) and specific primers, on a Light Cycler 96 Roche Instrument. Relative mRNA expression levels were calculated using cycle threshold (ΔΔCt) values. All values were normalized for the level of GAPDH mRNAs. All primer sequences are as follows. SOD2: (Forward (5′−3′): GCC TTC AAG TAT GAT CGG TTC CT, Reverse (5′−3′): GAT CTT CTT GCC CGA CTT GTA GA); PPARγ: (Forward (5′−3′): CCC AAT GGT TGC TGA TTA CA, Reverse (5′−3′): GGA CGC AGG CTC TAC TTT GA); Nrf2: (Forward (5′−3′): ACA TGG AGC AAG TTT GGC GGG, Reverse (5′−3′): TGG AGG ATG CTG CTG AAA); GCLC: (Forward (5′−3′): ACC ATC ATC AAT GGG AAG GA, Reverse (5′−3′): GCG ATA AAC TCC CTC ATC CA); NQO1: (Forward (5′−3′): TTA CTA TGG GAT GGG GTC CA, Reverse (5′−3′): TCT CCC ATT TTT CAG GCA AC); GAPDH: (Forward (5′−3′): GGT GAA GGT CGG TGA AG, Reverse (5′−3′): CTC GCT CCT GGA AGA TGG TG).

### Chromatin immunoprecipitation (ChIP)

AML-12 cells were seeded in 20 cm culture dishes and, after 24 h, were treated with CCl_4_ and DDB. After 24 h of treatment ChIP assays were performed using a ChIP Kit (Cat. abs50034, Absin, Shanghai, China) according to the manufacturer’s instructions. Antibody-immunoprecipitated DNA was analyzed via real time PCR and normalized to the input. The primer sequences are as follows: SOD2: (Forward (5′−3′): CTC TGG CTG TGA GCT GCA AAG CTT CCA C, Reverse (5′−3′): GGC TGA GTA GTT CCC GGT GGT TTT TTC C). PPARγ: (Forward (5′−3′): GCC TTA AGC AAG AAG CCA GAG TTT TCC TGA TTA C, Reverse (5′−3′): CGT GAA CTG TAC AGT AGT TGG AAT TAC CAG AGC).

### Molecular docking simulation

Molecular docking technology was employed to investigate the binding mode of DDB with Kelch-like ECH-associated protein 1 (Keap1). The crystal structure of Keap1 (PDB ID: 2DYH) was obtained from the Protein Data Bank (https://www.rcsb.org/). Furthermore, the structure of DDB (CID:442523) was sourced from the PubChem database (https://pubchem.ncbi.nlm.nih.gov/). The target protein and ligand compound were prepared according to standard protocols. Subsequently, these files were subjected to analysis using AutoDock.

### Drug affinity responsive target stability (DARTS) assay

AML-12 cells were harvested and lysed using 0.2% Triton lysis buffer, followed by centrifugation at 12,000 rpm for 10 min. The resultant lysates were then diluted with TNC buffer (comprising 50 mM Tris–HCl (pH 8.0), 50 mM NaCl, and 10 mM calcium chloride). The lysate was divided into four aliquots, which were each treated with DMSO or DDB (1 μM) at RT for 1 h. Post-incubation, the samples were further treated with streptomyces pronase (2 μg/ml, TargetMol, T13827) at RT for 40 min. Ultimately, 5X loading buffer was added to each sample, mixed thoroughly, and boiled at 100 ℃ for 10 min to denature proteins. The expression of Keap1 was subsequently assessed via WB analysis. The expression level of the target protein was represented by the ratio of the grayscale value of the target protein to that of the DMSO group.

### Cellular thermal shift assay (CETSA)

The AML-12 cells were collected and resuspended in PBS containing 1% protease inhibitors. Cell lysis was then carried out through five cycles of freezing and thawing. The resultant cell lysates were centrifuged at 12000 g for 10 min at 4 ℃ to collect the supernatant. The supernatant was divided equally into two portions, which were then treated with either 1 μM DDB or DMSO at RT for 1 h. Subsequently, the samples incubated with DDB or DMSO were further divided into ten aliquots and heated at temperatures of RT, 37, 41, 45, 49, 53, 57, 61, 65, and 69 ℃ for 3 min each. Following heating, the samples were centrifuged again at 12000 g for 10 min at 4 ℃ to harvest the supernatants, which were subsequently boiled with sample loading buffer. The expression of Keap1 was subsequently assessed via Western blot analysis.

### Determination of mtROS level

To assess the level of superoxide produced by mitochondria within cells, we use the MitoSOX Red kit (ThermoFisher) to quantify mtROS. First, cells are seeded in a 24-well plate containing coverslips and after 24 h, subjected to drug intervention. After an additional 24 h of treatment, cells are stained with 500 nM MitoSOX Red for 30 min. Following this, the cells on the coverslips are collected and fixed with tissue fixation solution, then stained with DAPI and mounted for observation. The content of mtROS is observed under confocal microscopy, and the mean fluorescence intensity is calculated from images taken at a 63 × magnification.

### Lentiviral transduction

To establish an Nrf2 overexpressing cell line, AML-12 cells were transduced with a lentiviral vector encoding Nrf2. The lentiviral vector was purchased from Hanbio Tech (Shanghai, China). Cells were seeded into 6-well plates, and when they reached a confluence of 30–40%, they were transfected with the lentivirus carrying the Nrf2 gene. After 48 h post-transfection, cells were selected with 5 µg/mL puromycin for 12 h. The selected cells underwent three rounds of screening to obtain stable Nrf2 overexpressing clones. Then, according to the previous procedure, cells were re-plated and exposed to 20 mM CCl_4_ for 24 h before being harvested for subsequent assays.

### Prediction of transcription factor binding sites

The NCBI gene database was used to search for the promoter regions of PPARγ and SOD2. The JASPAR database (https://jaspar.genereg.net) was utilized to analyze the binding sites of Nrf2 in the promoter region of PPARγ and SOD2, as well as the binding sites of PPARγ in the promoter region of SOD2.

### Luciferase reporter assay

DNA fragments containing the PPARγ binding sequence (pcDNA-Pparg) and its corresponding negative control sequence (pcDNA-NC) were designed, synthesized, and cloned into a luciferase reporter vector. The constructed reporter plasmids were co-transfected with either the wild-type (SOD2-WT) or mutant (SOD2-MT) SOD2 reporter vector into 293 T cells. Relative luciferase activity was measured using a dual-luciferase reporter assay system. Firefly luciferase activity was normalized to Renilla luciferase activity, and the results were used to evaluate the transcriptional regulation of SOD2 by PPARγ.

### Immunofluorescence staining

Cells were seeded onto coverslips placed in 12-well cell culture plates and cultured for 24 h before modeling and drug treatment. After 24 h of treatment, the culture medium was removed, and cells were washed with PBS. Cells were then fixed with 4% paraformaldehyde for 10 min, permeabilized with 0.1% Triton X-100 in PBS for 15 min, and blocked with 5% BSA in PBS for 1 h at room temperature. Primary antibody against PPARγ was applied and incubated overnight at 4 °C. The following day, cells were washed thoroughly with PBS and incubated with Alexa Fluor-labeled secondary antibody for 1 h at room temperature in the dark. Nuclei were counterstained with DAPI for 10 min. Coverslips were mounted onto glass slides using an anti-fade mounting medium and imaged under a fluorescence microscope.

### Statistical analysis

All experimental results are presented as mean ± standard error of mean (SEM), reflecting results from at least three independent experiments. Group differences were evaluated using the Student's t-test (two-tailed) and one-way analysis of variance (ANOVA), followed by Tukey's post hoc test for multiple comparisons. A *p*-value of < 0.05 was considered statistically significant.

## Results

### ***DDB mitigates CCl***_***4***_***-induced ALI in mice by modulating necroptosis***

**Fig. 1:**
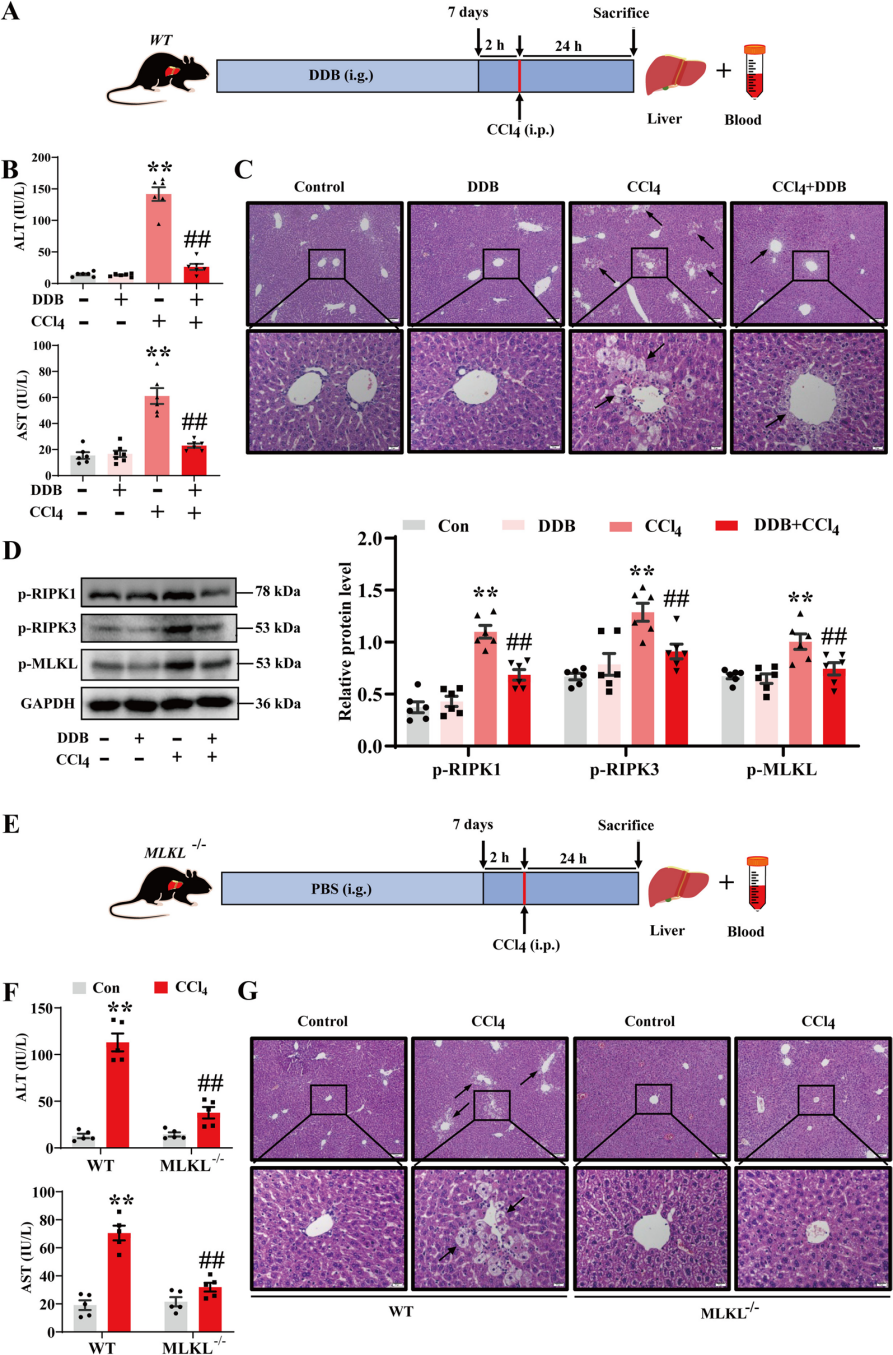
DDB alleviates CCl_4_-induced ALI in mice via modulation of necroptotic signaling. **A** The administration regimen for WT mice. **B** Serum levels of AST and ALT in WT mice (n = 6). **C** Representative H&E-stained sections of liver tissue from WT mice. Scale bars: 50 µm (top); 20 µm (bottom). **D** Expression levels and quantitative analysis of p-RIPK1, p-RIPK3 and p-MLKL proteins in liver tissues (n = 6). **E** The administration regimen for MLKL^−/−^ mice. **F** Serum AST and ALT levels in MLKL^−/−^ mice (n = 5). **G** Representative H&E-stained liver sections from MLKL^−/−^ mice. Scale bars: 50 µm (top); 20 µm (bottom). Compared with the control group, *P < 0.05, **P < 0.01; compared with the CCl_4_ group, ^#^*P* < 0.05, ^##^*P* < 0.01

This study evaluated the pharmacological efficacy of DDB against CCl_4_-induced hepatic toxicity in vivo (Fig. 1A). The results showed that, compared to the model group, DDB significantly reduced serum levels of liver function markers ALT and AST (Fig. 1B). Histological examination using H&E staining showed decreased hepatocellular enlargement and necrosis after DDB treatment (Fig. 1C), consistent with a protective effect against CCl_4_-induced ALI. Building on previous findings that MLKL-mediated necroptosis is involved in CCl_4_ induced induces ALI [9], this study explored DDB’s mechanism in inhibiting necroptosis. Exposure to CCl_4_ upregulated RIPK1, RIPK3, and MLKL protein expression (Fig. S1A) and their phosphorylated forms (Fig. 1D). DDB administration significantly suppressed CCl_4_-induced phosphorylation of MLKL, although levels remained elevated compared to the control group. To further characterize necroptosis involvement, MLKL^−/−^ mice were used (Fig. 1E). Compared to the WT model group, CCl_4_-treated MLKL^−/−^ mice showed significantly lower serum ALT and AST levels (Fig. 1F) and improved liver tissue necrosis and hepatocyte swelling (Fig. 1G), this is similar to the effect of DDB. Collectively, these findings support a role for DDB in attenuating CCl_4_-induced ALI, potentially through modulation of necroptosis.

### DDB rescues hepatic injury through Nrf2, PPARγ, and SOD2-related pathways

**Fig. 2:**
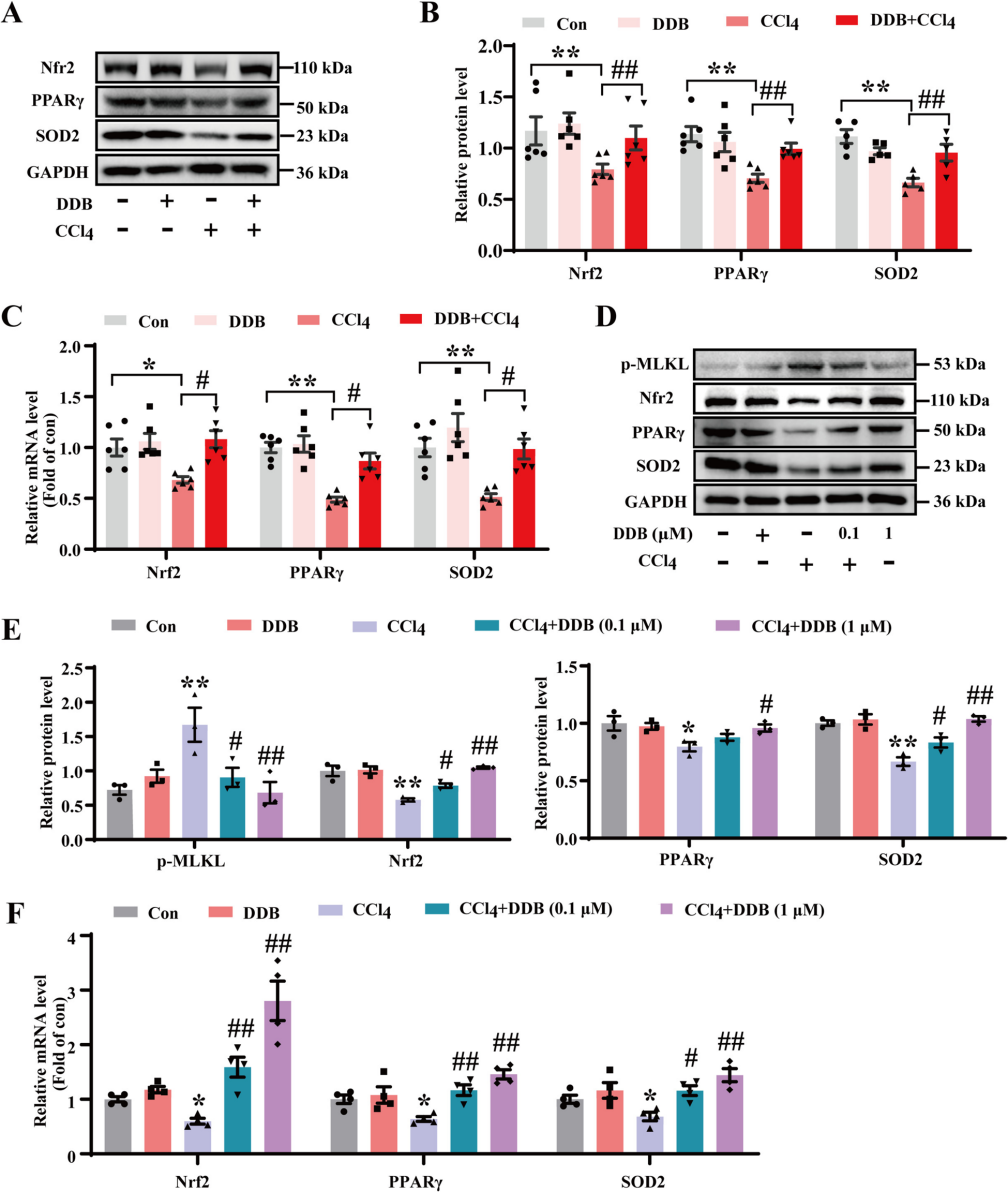
DDB affects expression levels of Nrf2, PPARγ, and SOD2. **A** Expression levels of Nrf2, PPARγ, and SOD2 proteins in liver tissues. **B** Quantitative analysis of Nrf2, PPARγ (n = 6), and SOD2 (n = 5). **C** mRNA expression of Nrf2, PPARγ, and SOD2 in tissue samples (n = 6). **D** Expression levels of p-MLKL, Nrf2, PPARγ, and SOD2 proteins in AML-12 cells. **E** Quantitative analysis of p-MLKL, Nrf2, PPARγ, and SOD2 (n = 3). **F** mRNA expression of Nrf2, PPARγ, and SOD2 in AML-12 cells (n = 4). Compared with the control group, *P < 0.05, **P < 0.01; compared with the CCl_4_ group, ^#^*P* < 0.05, ^##^*P* < 0.01

Studies have demonstrated that DNLA exerts hepatoprotective effects by activating the Nrf2 pathway and regulating oxidative stress [9], but the precise mechanism was unclear. This study investigated the impact of DDB on Nrf2, PPARγ, and SOD2. Results showed that CCl_4_ treatment decreased the expression of these proteins, while DDB administration upregulated their expression (Fig. 2A and B). Similarly, mRNA levels of Nrf2, PPARγ, SOD2, GCLC and NQO1 were reduced by CCl_4_ exposure and increased by DDB (Fig. 2C and Fig. S2A). To further explore the underlying mechanism, AML-12 cells were used in vitro. The results validated the in vivo findings, showing that DDB treatment reduced the expression level of necroptosis executor p-MLKL, which was elevated by CCl_4_, thereby inhibiting necroptosis (Fig. 2D and E). In addition, DDB prevented the CCl_4_-induced decreases in the mRNA levels of Nrf2, PPARγ, and SOD2 (Fig. 2F). These findings suggest that DDB may protect CCl_4_-induced ALI by modulating the protein and mRNA expression of Nrf2, PPARγ and SOD2.

### *DDB ameliorates cell damage by upregulating SOD2 *via* PPARγ and suppressing necroptosis*

**Fig. 3:**
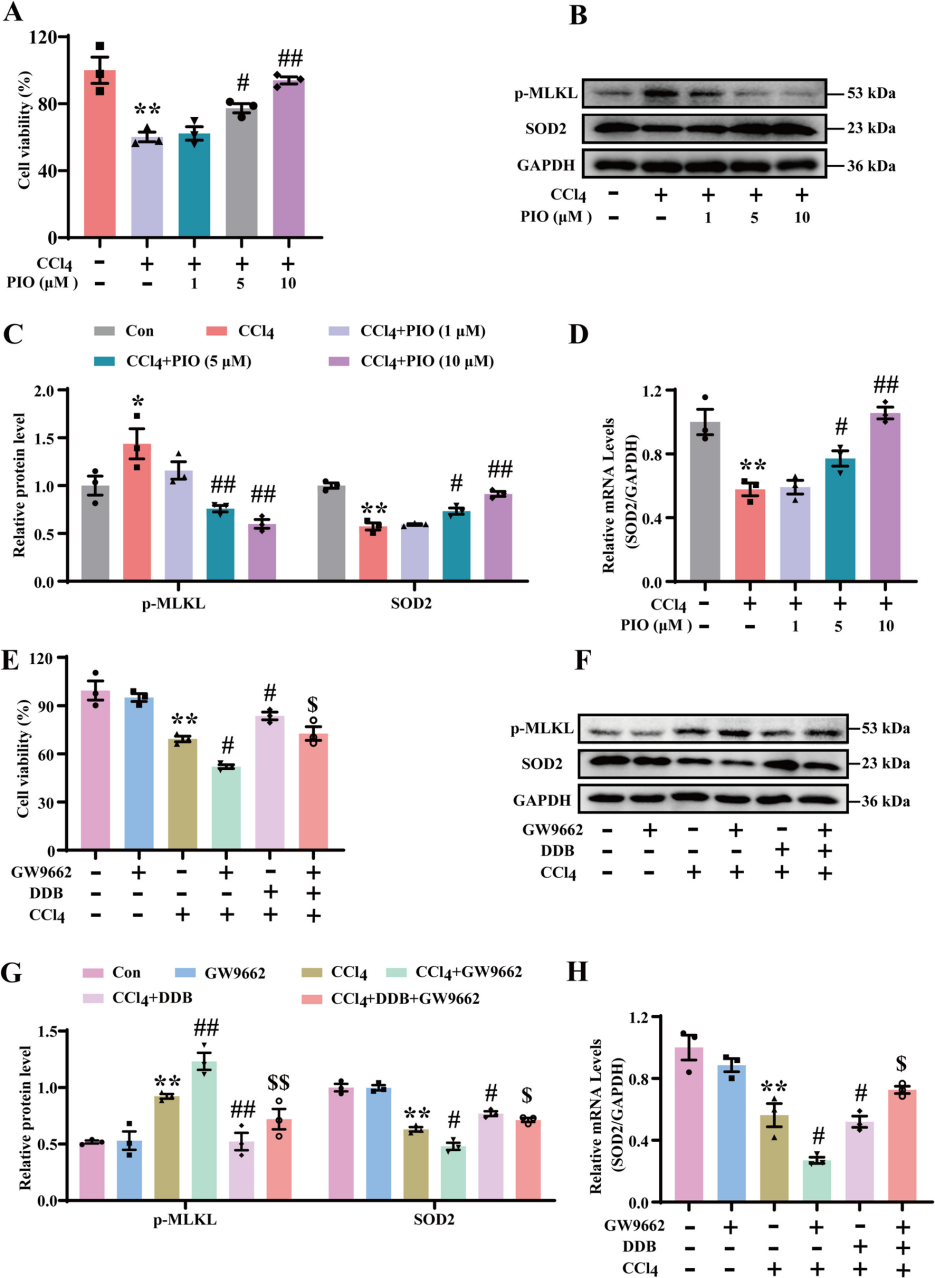
DDB inhibits AML-12 cell damage by regulating SOD2 via PPARγ and suppressing necroptosis. **A** The effect of PIO on cell viability of AML-12 cells subjected to CCl_4_-induced injury (n = 3). **B** Expression levels of p-MLKL and SOD2 proteins in AML-12 cells. **C** Quantitative analysis of p-MLKL and SOD2 (n = 3). **D** mRNA expression of SOD2 in AML-12 cells. (n = 3). **E** The effect of GW9662 on cell viability of AML-12 cells subjected to CCl_4_-induced injury (n = 3). **F** Expression levels of p-MLKL and SOD2 proteins in AML-12 cells. **G** Quantitative analysis of p-MLKL and SOD2 (n = 3). **H** mRNA expression of SOD2 in AML-12 cells. (n = 3). Compared with the control group, *P < 0.05, **P < 0.01; compared with the CCl_4_ group, ^#^*P* < 0.05, ^##^*P* < 0.01; compared with the GW9662 + CCl_4_ group, ^$^*P* < 0.05, ^$$^*P* < 0.01

The literature indicates that PPARγ, a nuclear transcription factor, is involved in modulating the expression of genes associated with oxidative stress [30, 31]. In the present study, AML-12 cells were pre-treated with the PPARγ agonist pioglitazone (PIO) prior to stimulation with CCl_4_. As the concentration of PIO increased, cell viability improved (Fig. 3A), and the level of the necroptotic executor protein p-MLKL decreased (Fig. 3B). Concurrently, the protein and mRNA levels of SOD2 increased (Fig. 3C and D). To explore whether DDB inhibits necroptosis via PPARγ upregulation, this study used GW9662, a PPARγ inhibitor, in vitro. GW9662 exacerbated CCl_4_-induced injury and reduced viability in AML-12 cells, while these effects were mitigated by DDB treatment (Fig. 3E). Compared to the CCl_4_ group, GW9662 further increased the CCl_4_-induced protein expression levels of p-MLKL (Fig. 3F) and decreased SOD2 protein and mRNA levels (Fig. 3G and H). In contrast, DDB administration decreased p-MLKL protein expression and increased SOD2 protein and mRNA levels (Fig. 3G and H). In summary, DDB may suppress necroptosis and alleviate hepatic injury by upregulating PPARγ, which in turn regulates SOD2 expression.

### ***Inhibition of PPARγ aggravates CCl***_***4***_***-induced ALI by exacerbating necroptosis ***in vivo

**Fig. 4:**
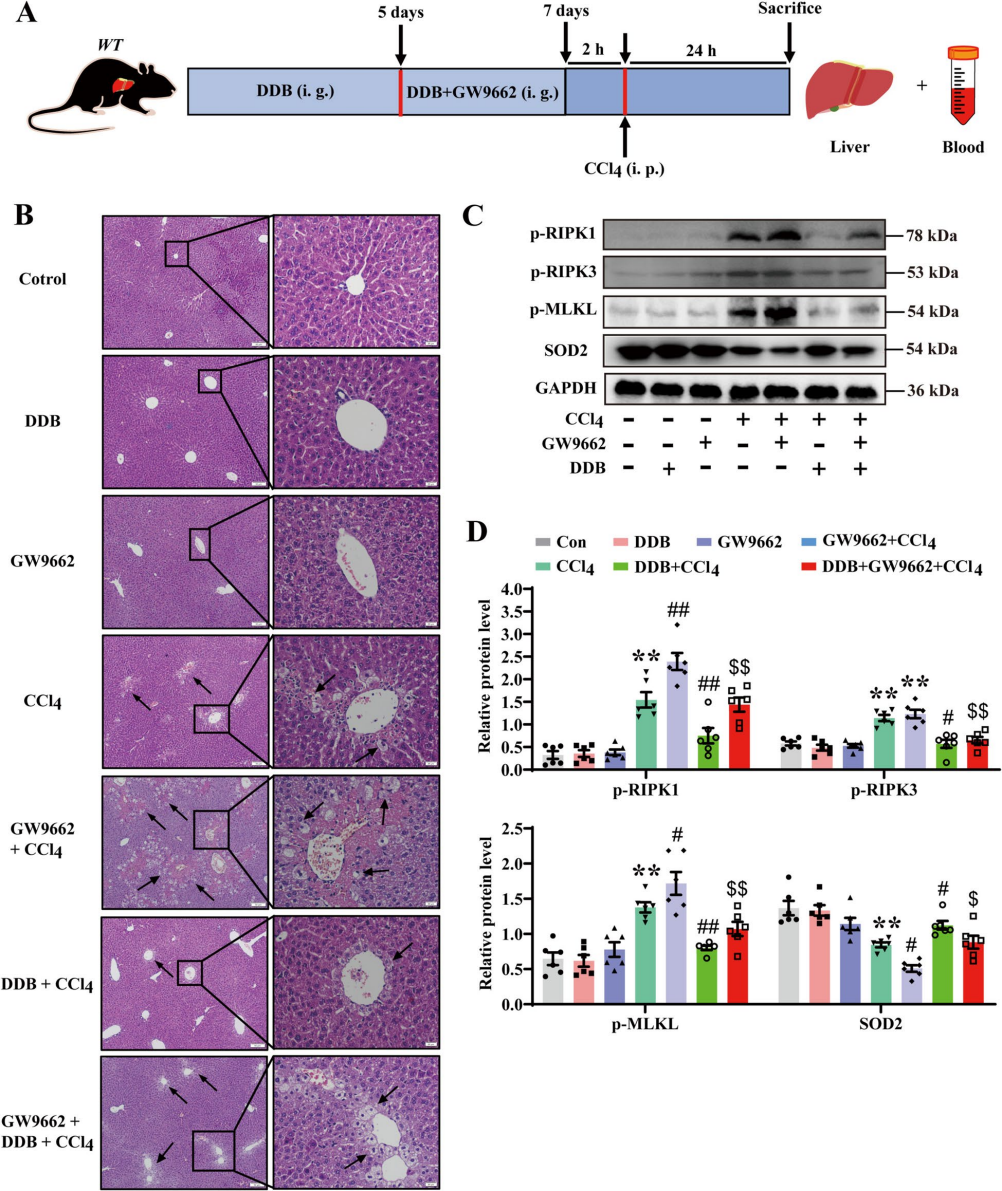
In vivo inhibition of PPARγ exacerbates CCl_4_-induced ALI via necroptosis. **A** Experiment design. **B** Representative H&E-stained liver sections from mice treated with GW9662. Scale bars: 50 µm (top); 20 µm (bottom). **C** Expression levels of p-RIPK1, p-RIPK3, p-MLKL, and SOD2 proteins in liver tissues following GW9662 administration. **D** Quantitative analyses of p-RIPK1, p-RIPK3, p-MLKL, and SOD2 (n = 6). (n = 6) Compared with the control group, *P < 0.05, **P < 0.01; compared with the CCl_4_ group, ^#^*P* < 0.05, ^##^*P* < 0.01; compared with the GW9662 + CCl_4_ group, ^$^*P* < 0.05, ^$$^*P* < 0.01

To investigate whether the protective effect of DDB is mediated via the PPARγ/SOD2 axis, the PPARγ antagonist GW9662 was administered to mice (Fig. 4A). Results showed that compared to CCl_4_-only treatment, GW9662 significantly increased serum ALT/AST and worsened hepatocellular injury. In contrast, DDB treatment reduced ALT and AST levels (Fig. S3A) and attenuated histological signs of liver injury (Fig. 4B), indicating a protective effect even in the presence of PPARγ inhibition. Additional analysis of RIPK1/RIPK3/MLKL phosphorylation levels showed that, compared to the CCl_4_ group, administration of GW9662 significantly increased the phosphorylation levels of RIPK1/RIPK3/MLKL, indicating exacerbated necroptosis. Notably, concurrent administration of DDB reduced these levels (Fig. 4C and D). In vivo*,* the administration of GW9662 also reduced the protein and mRNA expression of SOD2, which was rescued by DDB (Fig. S3B). These in vivo observations are consistent with the in vitro findings, supporting a role for PPARγ in regulating SOD2 expression, which may contribute to the inhibition of necroptosis by DDB.

### DDB enhances transcription and translation of SOD2 by upregulating PPARγ expression to clear mtROS

**Fig. 5:**
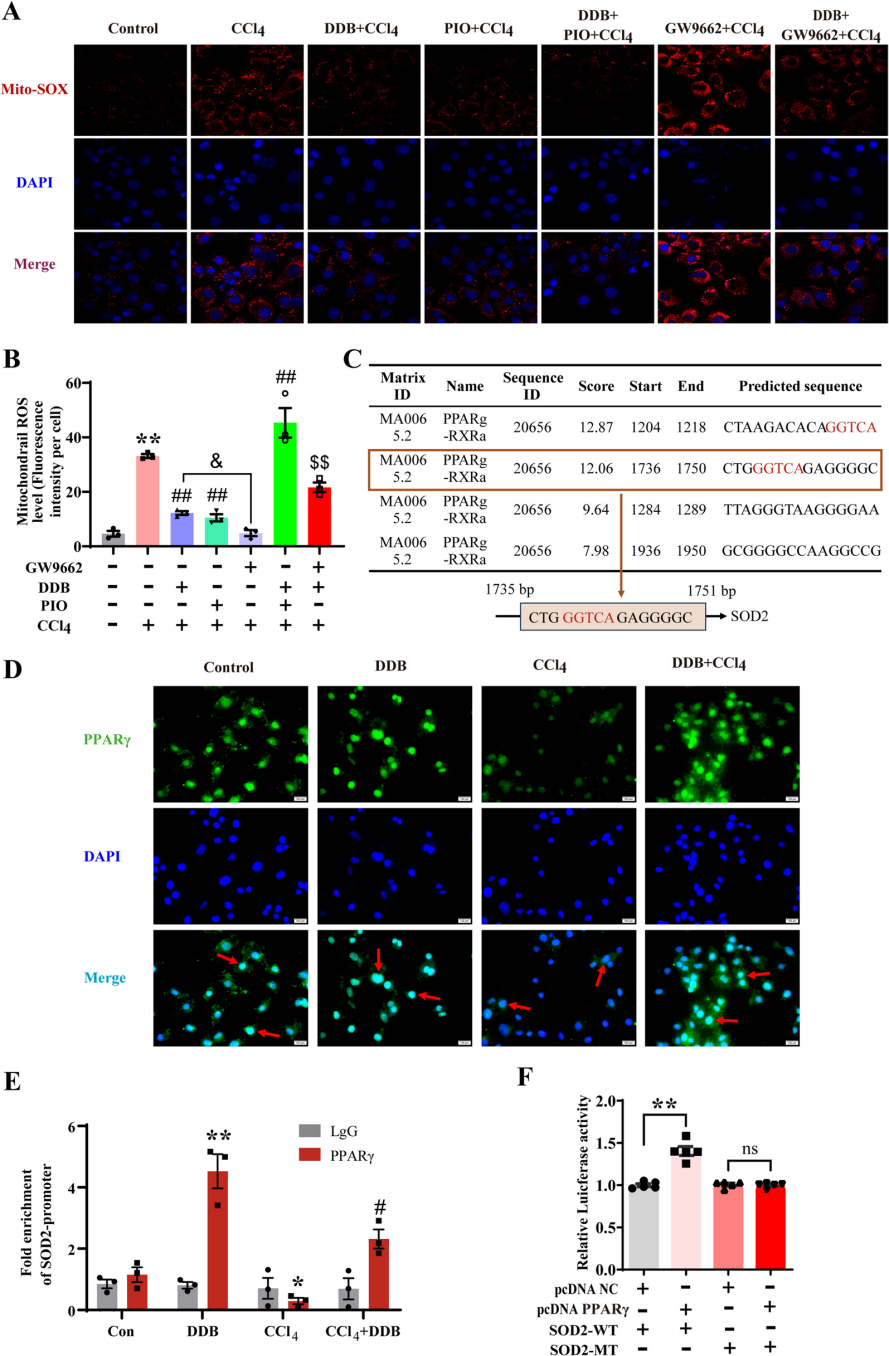
DDB enhances transcription and translation of SOD2 by upregulating PPARγ expression and clears mtROS. **A-B** Impact of PPARγ on mtROS levels in AML-12 cells (n = 3). **C** The predicted binding sites for PPARγ and SOD2, with red letters denoting the PPRE. **D** Immunofluorescence analysis of PPARγ nuclear translocation (representative images of n = 3 independent experiments). **E** Fold enrichment of the SOD2 promoter region (n = 3). **F** Relative luciferase activity of SOD2 in response to PPARγ (n = 5). Compared with the control group, *P < 0.05, **P < 0.01; compared with the CCl_4_ group, ^#^*P* < 0.05, ^##^*P* < 0.01; compared with the GW9662 + CCl_4_ group, ^$$^*P* < 0.01

Previous research from our group demonstrated that DNLA reduces mtROS and inhibits CCl_4_-induced necroptosis, and overexpression of SOD2 similarly inhibits necroptosis activation by scavenging mtROS [9]. In this study, in vitro results showed that PIO can significantly reduce the CCl_4_-induced elevation of mtROS levels. In contrast, intervention with GW9662 resulted in a significant increase in mtROS, which was mitigated by DDB treatment (Fig. 5A and B). These findings suggest that DDB may affect mtROS levels and necroptosis by regulating PPARγ, though the exact mechanism is unclear. Using the JASPAR online tool for prediction, we found that the promoter sequence of SOD2 contains the PPARγ response element (PPRE) (Fig. 5C), suggesting that PPARγ may regulate the SOD2 promoter. Immunofluorescence showed that CCl_4_ led to a decrease in PPARγ expression and nuclear translocation, while DDB administration increased both PPARγ expression and nuclear translocation (Fig. 5D). Further, ChIP assays revealed that DDB increased the binding of PPARγ to the SOD2 promoter (Fig. 5E). The dual luciferase assay indicated that PPARγ increases the relative luciferase activity of the SOD2 promoter in 293 T cells, and this effect disappeared when the promoter sequence of SOD2 was mutated (Fig. 5F), further confirming the direct regulation of SOD2 by PPARγ. These results indicate that DDB upregulates PPARγ expression, thereby increasing the transcription of SOD2, clearing mtROS, and inhibiting necroptosis.

### *DDB regulates PPARγ and SOD2 expression *via* Nrf2 to suppress necroptosis*

**Fig. 6:**
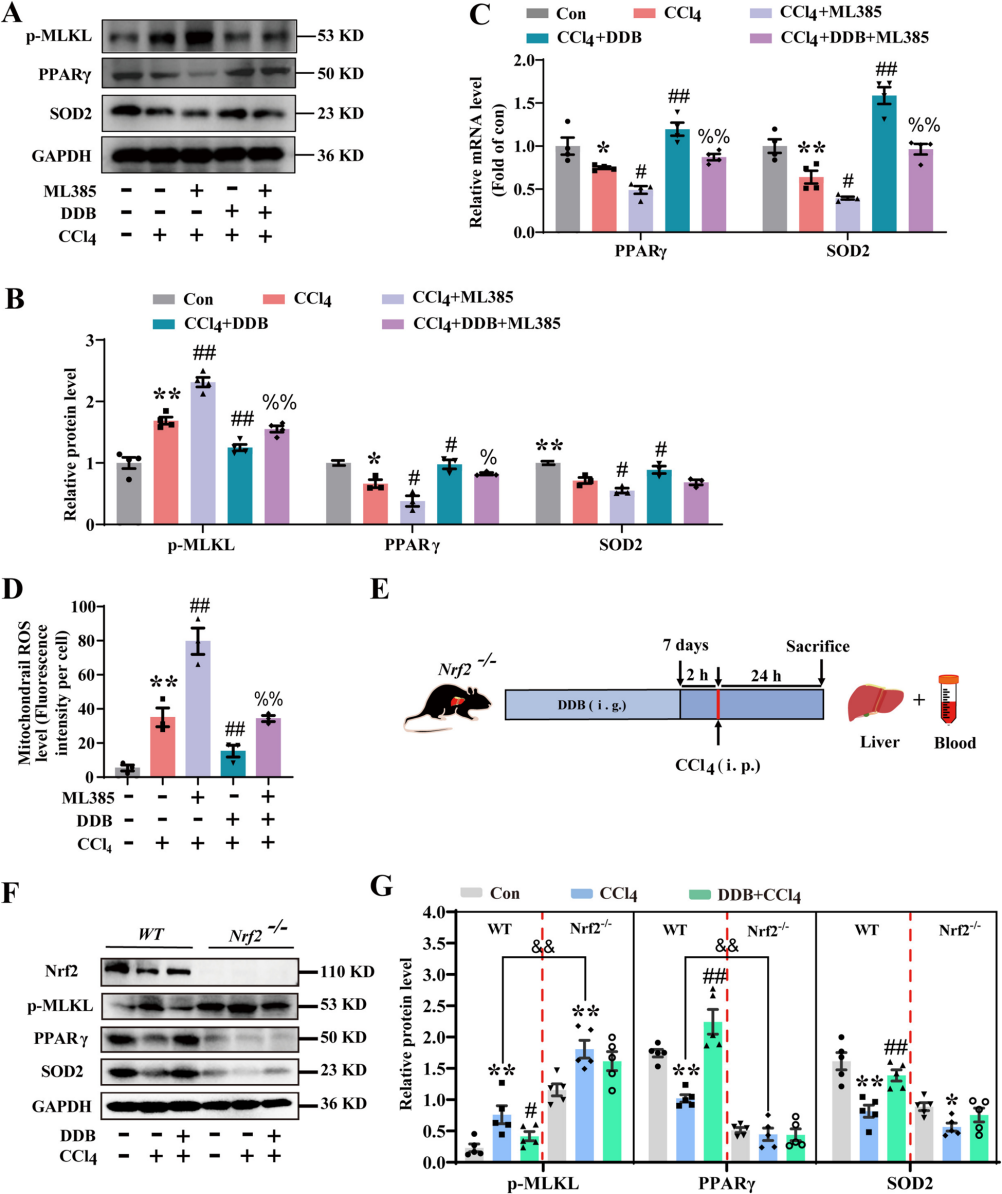
DDB regulates PPARγ and SOD2 expression via Nrf2 to suppress necroptosis. **A** Expression levels of p-MLKL, PPARγ and SOD2 proteins in AML-12 cells.** B** Quantitative analysis of p-MLKL, PPARγ and SOD2 (n = 3). **C** mRNA expression of PPARγ and SOD2 in AML-12 cells. (n = 3). **D** Impact of Nrf2 on mtROS levels in AML-12 cells (n = 3). **E** Experiment design. **F** Expression levels of Nrf2, p-MLKL, PPARγ and SOD2 proteins in WT and Nrf2^−/−^ mice. **G** Quantitative analysis of p-MLKL, PPARγ and SOD2 (n = 5). Compared with the control group, *P < 0.05, **P < 0.01; compared with the CCl_4_ group, ^#^*P* < 0.05, ^##^*P* < 0.01; compared with the ML385 + CCl_4_ group, ^%^*P* < 0.05, ^%%^*P* < 0.01

Previous research has established that DNLA regulates oxidative stress via Nrf2, thereby protecting mitochondrial function and safeguarding the liver from damage. In this study, when the Nrf2 inhibitor ML385 was administered, the expression of p-MLKL increased (Fig. 6A and B), while the protein and mRNA levels of PPARγ and SOD2 decreased (Fig. 6B and C), and mtROS levels significantly increased (Fig. 6D and S4A). However, administration of DDB markedly reversed these changes. These observations suggest that DDB may regulate the expression of PPARγ and SOD2 through Nrf2, thereby modulating necroptosis. To further test our hypothesis, we used Nrf2^−/−^ mice (Fig. 6E and F). The results showed that after Nrf2 knockout, the expression of PPARγ and SOD2 was significantly reduced, and the expression of p-MLKL was further increased (Fig. 6G and H). However, the protective effect of DDB was abrogated. These findings indicate that Nrf2 is indispensable for DDB-mediated suppression of necroptosis, and its absence severely compromises the protective effects of DDB. Collectively, these data suggest that DDB may attenuate mtROS accumulation and necroptosis through an Nrf2-dependent mechanism involving the regulation of PPARγ and SOD2.

### Nrf2 directly regulates the expression of PPARγ

**Fig. 7:**
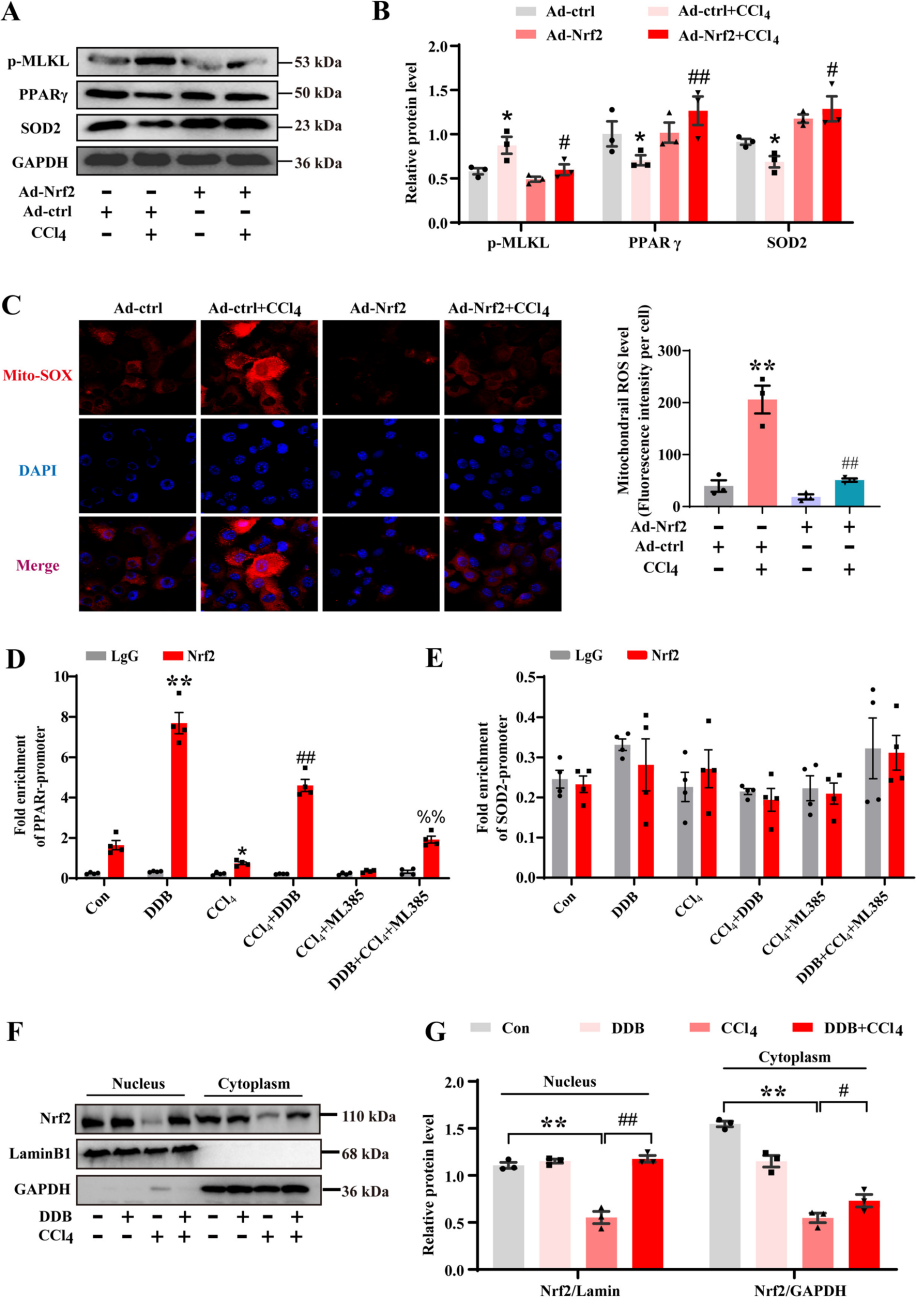
Nrf2 directly regulates the expression of PPARγ. **A** Expression levels of p-MLKL, PPARγ and SOD2 proteins in overexpression of Nrf2 cells. **B** Quantitative analysis of p-MLKL, PPARγ and SOD2 (n = 3). **C** Effect of CCl_4_ exposure on mtROS levels in Nrf2 overexpressing cells (n = 3). **D-E** ChIP assay for PPARγ and SOD2 promoter binding with Nrf2 in AML-12 cells (n = 4). **F-G** WB was applied to detect cytoplasmic and nuclear expression of Nrf2. Lamin B1 and GAPDH were used to prove the protein integrity of nucleus and cytoplasm respectively (n = 3). Compared with the control group, *P < 0.05, **P < 0.01; compared with the CCl_4_ group, ^#^*P* < 0.05, ^##^*P* < 0.01; compared with the ML385 + CCl_4_ group, ^%%^*P* < 0.01

To further validate our hypothesis, AML-12 cells were transduced with a lentiviral vector encoding Nrf2 (Fig. S5A-B), followed by CCl_4_ and DDB administration. The results showed that the overexpression of Nrf2 inhibited the toxicity of CCl_4_ compared to the model group with the control vector. The protein expression of PPARγ and SOD2 did not decrease further, and p-MLKL did not increase (Fig. 7A and B). Meanwhile, overexpression of Nrf2 significantly reduced mtROS in the model group (Fig. 7C). Given Nrf2's influence on PPARγ and SOD2, we speculated that their promoter regions may contain an antioxidant response element (ARE) for transcriptional activation. Analysis of the murine PPARγ 5′ flanking region identified a potential ARE located at positions − 1258/− 1248 upstream of the transcription start site (Fig. S5C). However, no potential ARE binding sequences were found in the SOD2 promoter. ChIP assays confirmed that DDB significantly enhanced Nrf2 binding to the PPARγ promoter (Fig. 7D). Notably, it was found that Nrf2 does not bind to the SOD2 promoter (Fig. 7E). This suggests that DDB regulates the PPARγ promoter via Nrf2, subsequently influencing SOD2 expression, rather than directly regulating SOD2. Concurrently, quantification of nuclear Nrf2 protein levels revealed that DDB increased Nrf2’s nuclear accumulation (Fig. 7F and G), further substantiating its ability to modulate the PPARγ promoter. Collectively, these findings indicate that Nrf2 can directly regulate the expression of PPARγ, and DDB can suppress necroptosis and alleviate hepatocyte damage by activating Nrf2.

### DDB binds to the Kelch structural domain of Keap1

**Fig. 8:**
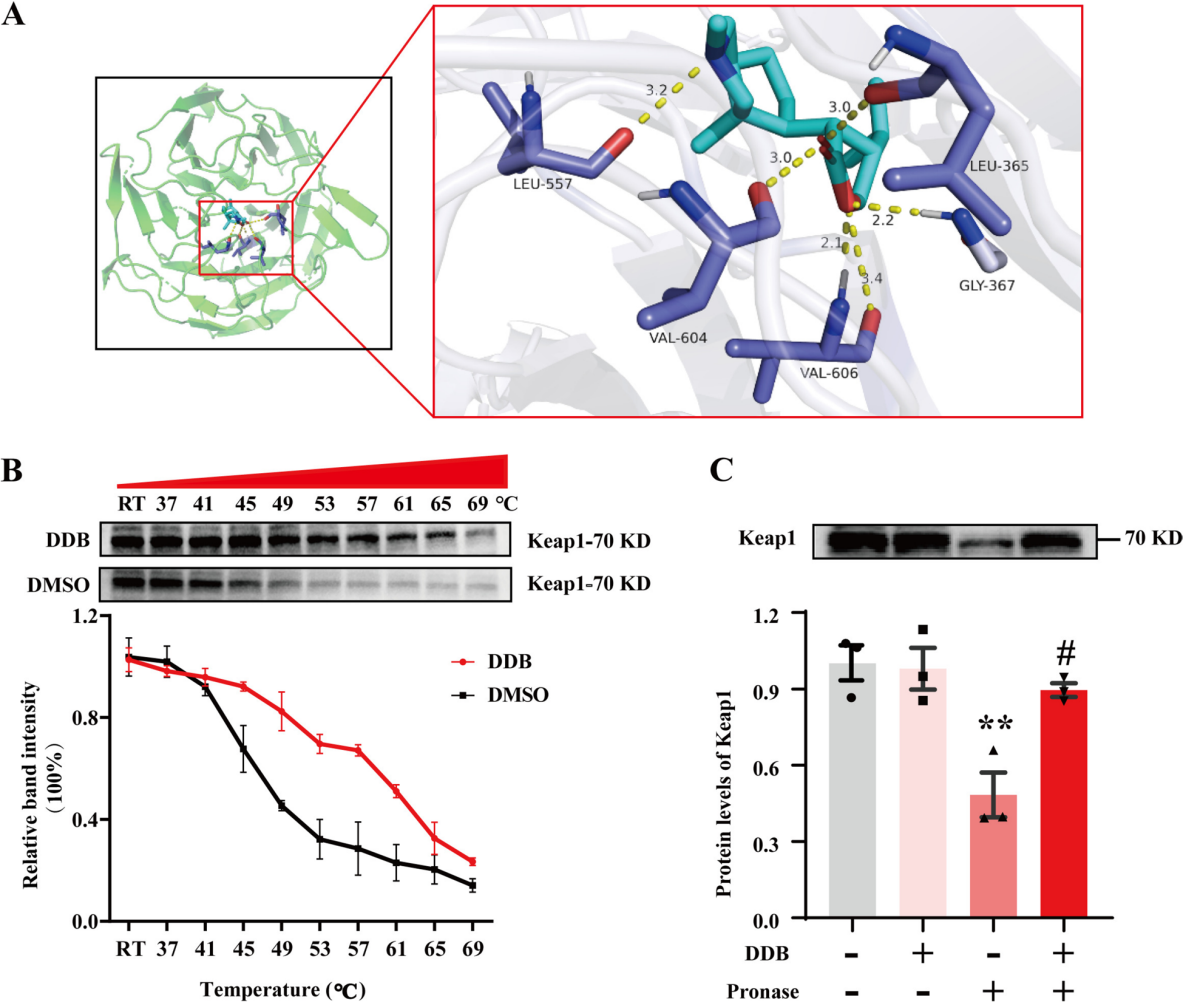
DDB binds to the Kelch structural domain of Keap1. **A** The DDB binding sequence was located within the Kelch domain of Keap1. **B** The interaction between DDB and Keap1 was explored by means of the CETSA assay (n = 3). **C** Western blot analysis showed that DDB enhanced the resistance of Keap1 to proenzyme digestion in AML-12 cells (n = 3). Compared with the control group, **P < 0.01; compared with the pronase group, ^#^*P* < 0.05

According to the introduction, Nrf2 must dissociate from Keap1 before it can be translocated into the nucleus, which is also the primary mechanism of action for most Nrf2 activators. To elucidate how DDB enhances Nrf2 activation, we investigated the interaction between DDB and the Nrf2-binding domain (Kelch domain) of Keap1 using molecular docking simulations. Results showed that DDB binds tightly to this domain with a binding energy of − 7.4 kcal/mol, primarily through hydrogen bonds with Leu365, Gly367, Leu557, Val604, and Val606 (Fig. 8A). The cellular thermal shift assay (CETSA) confirmed DDB’s interaction with Keap1, evidenced by stronger immunoblot bands in DDB-treated cells compared to DMSO controls across temperatures from 49 °C to 69 °C (Fig. 8B). Further investigation using the drug affinity responsive target stability (DARTS) method revealed that DDB significantly stabilized Keap1 against streptomycin-induced degradation (Fig. 8C). These findings suggest that DDB robustly binds to the Kelch domain of Keap1, releasing Nrf2 and enhancing its nuclear translocation. This mechanism enhances the transcriptional and translational control of PPARγ, coordinating SOD2 expression to mitigate mtROS and suppressing necroptosis, thereby exerting a hepatoprotective effect.

## Discussion

Necroptosis has been increasingly recognized to play a critical role in various liver injuries [9, 32]. In this study, we described a Nrf2/PPARγ/SOD2 axis and its role in the regulation of necroptosis, and determined that DDB is a hepatoprotective agent that can inhibit MLKL activation*,* thereby inhibiting necroptosis and improving ALI. The hepatoprotective mode of action of DDB was mediated by regulating the Nrf2/PPARγ/SOD2 pathway through Keap1. These findings were validated through both in vitro and in vivo experiments*.* Specifically, DDB facilitated the activation and nuclear translocation of Nrf2 by binding to Keap1, resulting in the accumulation of Nrf2 in the nucleus, and upregulation of PPARγ and SOD2. Importantly, we demonstrated that Nrf2 directly activated PPARγ rather than SOD2, and the upregulation of SOD2 was achieved through the direct transcriptional regulation by PPARγ. The upregulation of PPARγ led to increased SOD2 expression that reduced mtROS generation and inhibited the activation of MLKL, thereby ameliorating necroptosis and alleviating liver injury. These findings provide evidence for the potential use of DDB in the prevention, treatment, and improvement of ALI.

It is worth noting that the dose of DDB (20 mg/kg) used in this study was selected based on previous findings demonstrating its dose-dependent protective effects against hepatic steatosis at doses of 5–15 mg/kg [29], as well as our group’s observation that *Dendrobium nobile* Lindl. alkaloids (DNLA)—in which DDB accounts for 90.7%—exert significant protection against CCl_4_-induced acute liver injury at 20 mg/kg [9]. Furthermore, DDB has been shown to be well-tolerated with no obvious toxicity at doses up to 40 mg/kg [26], supporting the safety profile of the selected dose. Considering these dose–response relationships, 20 mg/kg was chosen as the effective dose for investigating the hepatoprotective mechanism of DDB in this study.

The role of necroptosis in liver injury represents a complex and active area of research. MLKL is a primary executor of the necroptotic pathway. The activation of MLKL is a necessary condition for necroptosis [4], but the mechanism of its activation is complex and has not yet been fully elucidated. It is generally believed that ROS is a critical player in the activation of necroptosis. Studies in recent years have demonstrated that PPARγ activation can inhibit necroptosis by regulating MLKL [33, 34]. However, the specific role and molecular mechanism of PPARγ in necroptosis are largely unclear, especially with little research on PPARγ dependent regulation of oxidative status in the context of necroptosis. One of important findings of the present study involves the characterization of the role of PPARγ-regulated SOD2 in necroptosis, and the activation of PPARγ/SOD2 signaling is directly related to the protective mechanism of DDB on liver injury. Our previous research showed that inhibiting mtROS can suppress CCl_4_-induced necroptosis [9]. In this study, we provided more evidence that mtROS participates in necroptosis by directly activating MLKL, whereas MLKL^−/−^ mice exhibits a heightened resistance to ALI induced by CCl_4_. In addition, we demonstrated that manipulating mtROS alters the phosphorylation of MLKL, i.e. activation of PPARγ reduces mtROS, thereby lowering p-MLKL levels and inhibiting necroptosis. Furthermore, we found that both in vitro and in vivo PPARγ inhibition resulted in an exacerbation of necroptosis and downregulation of SOD2. On the other hand, activation of PPARγ prevented CCl_4_-induced activation of necroptosis and downregulation of SOD2 expression. Importantly, we demonstrated that the expression SOD2 is transcriptionally regulated by PPARγ, under the scenario of CCl_4_-necroptosis. These results indicate that PPARγ is involved in the mechanism of necroptosis through regulating the expression of SOD2.

Studies have shown that DNLA has significant potential in preventing CCl_4_-induced ALI by reducing oxidative stress and inflammation, activating the Nrf2 signaling pathway, and inhibiting hepatocyte necroptosis [22, 35]. Notably, the primary component of DNLA is thought to be DDB [9, 36], which is hypothesized to be the key component responsible for the observed hepatoprotective effects. This study demonstrated that DDB exhibits a comparable liver protective effect to DNLA. In vivo and in vitro studies revealed that CCl_4_ induces upregulation of p-MLKL, leading to necroptosis activation, along with downregulation of PPARγ and SOD2 at both the protein and mRNA levels. Treatment with DDB upregulated PPARγ and SOD2 expression, thereby downregulating p-MLKL and inhibiting necroptosis. Previous studies have reported that DDB can activate PPARα and its downstream genes to exert hypolipidemic effects and improve NAFLD [29]. However, whether DDB can influence necroptosis through the regulation of PPARγ and SOD2 has not been reported. In this study, treatment with different concentrations of PPARγ agonist PIO in vitro dose-dependently blocked CCl_4_-induced downregulation of SOD2 expression and MLKL activation. Similarly, mtROS was also inhibited by PIO, with an effect comparable to that of DDB, while the protective effects of DDB were significantly inhibited by PPARγ antagonist GW9662 both in vivo and in vitro. Importantly, we identified SOD2 as a direct target of PPARγ through the JASPAR database and confirmed this by ChIP assays, showing that PPARγ can directly bind to the SOD2 promoter to regulate its transcription. In summary, DDB exerts its hepatoprotective effects by modulating the PPARγ/SOD2 signaling pathway, inhibiting mtROS, and thus suppressing necroptosis.

Nrf2 plays a pivotal role in the protection of the liver. In most cases, the activation of Nrf2 has been demonstrated to alleviate liver injury [22, 23]. The recent evidence indicates that the down-regulation of Nrf2 intensifies necroptosis in liver injury [37], whereas the activation of nuclear translocation of Nrf2 stimulates the expression of its downstream antioxidant enzymes, thereby exerting a protective role through the regulation of necroptosis [38, 39]. Nevertheless, the precise function of Nrf2 following nuclear translocation and the targets of antioxidant proteins it regulates remain to be fully elucidated in the context of necroptosis. A principal outcome of this study is the delineation of the functional role in the necroptosis. Furthermore, this study provides evidence that there is a direct relationship between Nrf2 activation and the protective mechanisms of DDB against liver injury. In vitro, the treatment of AML-12 cells with Nrf2 inhibitor ML385 resulted in an increase in mtROS, a reduction in PPARγ and SOD2 expression, and a reversal of the DDB-induced down-regulation of p-MLKL. These findings confirmed the role of Nrf2 in necroptosis and demonstrated its association with PPARγ and SOD2. In vivo studies revealed a notable decline in PPARγ and SOD2 levels, accompanied by a pronounced elevation in p-MLKL, in Nrf2^−/−^ mice following CCl_4_ treatment, in comparison to WT mice. Notably, DDB treatment did not offer protection in this context. This further confirms the role of Nrf2 in the liver injury protective mechanism, which involves the regulation of the PPARγ/SOD2 pathway. Furthermore, the results of this study revealed that DDB could counteract CCl_4_-induced ALI by activating Nrf2 nuclear translocation. Similarly, in cells overexpressing Nrf2, the expression of PPARγ and SOD2 did not decrease after CCl_4_ treatment, which was in stark contrast to wild-type cells with significantly reduced expression of PPARγ and SOD2. The overexpression of Nrf2 inhibited the increase of intracellular mtROS and the expression of the necrotic pore formation execution protein p-MLKL. This further confirms that DDB exerts its protective mechanism in liver injury through the regulation of Nrf2, involving the modulation of the PPARγ/SOD2 molecular signaling. Studies have reported that the activation of Nrf2 is closely associated with SOD2 and involves in the protective mechanism against various liver injuries [40, 41]. We have previously reported that Nrf2 activation leads to upregulation of SOD2, which plays an important role in liver protection in CCl_4_-induced ALI [22]. Despite these facts, how Nrf2 regulates SOD2 has not been fully defined yet. In the present study we demonstrated through ChIP experiments that SOD2 is not directly regulated by Nrf2 but by PPARγ, while Nrf2 directly regulates PPARγ. Indeed, previous studies have also reported that Nrf2 does not directly regulate SOD2, but rather does so indirectly through the action of other molecules and signaling pathways [42]. The present study further confirms this finding that Nrf2 directly regulates PPARγ but not SOD2, at least in the context of CCl_4_-induced necroptosis. Collectively, the data in the present work suggest that DDB protects against CCl_4_-induced ALI by inhibiting necroptosis through activating the Nrf2/PPAR/SOD2 pathway.

The Keap1-Nrf2 pathway represents a regulatory system that regulates the activity of Nrf2, a protein that is responsible for controlling the expression of antioxidants [43]. Keap1, originally identified by Itoh et al. through a yeast two-hybrid screening, serves as the chief repressor of Nrf2 under basal conditions [44]. The results in this study revealed that in CCl_4_-induced injury, the expression and nuclear translocation of Nrf2 protein were inhibited, whereas DDB significantly increased the nuclear translocation and protein expression of Nrf2. Furthermore, we found that the protective effect of DDB was significantly reduced when Nrf2 was knocked out. Therefore, we hypothesized that DDB exerts its hepatoprotective effects by binding to Keap1, releasing Nrf2 into the nucleus and activating the Nrf2/PPARγ signaling pathway. The structure of Keap1 can be divided into five distinct regions: (i) NTR domain (1–49 aa), (ii) BTB domain (50–179 aa), (iii) IVR domain (180–314 aa), (iv) DGR domain (327–611 aa), and (v) CTR domain (612–624 aa) [45, 46]. The DGR domain, also known as the Kelch domain, contains multiple protein-binding sites, including the protein contact site that mediates the interaction between Keap1 and Nrf2 [44, 47]. In this study, we performed docking experiments using the DGR region of Keap1 (324–618 aa), and the results suggest a possible interaction between Keap1 and DDB. Furthermore, CETSA and DARTS experiments collectively demonstrated that DDB can directly interact with Keap1, potentially disrupting the Keap1-Nrf2 association and releasing Nrf2 into the nucleus. This increased the transcription and translation of PPARγ and SOD2, thereby inhibiting mtROS production and reducing MLKL activation, which contributes to hepatoprotection. Although the specific interaction sites between DDB and Keap1 require further identification, this study provides substantial evidence that DDB directly targets Keap1, releasing Nrf2 into the nucleus to activate the PPARγ/SOD2 signaling pathway, inhibit necroptosis, and exert hepatoprotective effects.

Identification of the role and regulatory mechanism of PPARγ in necroptosis extends our understanding of Nrf2 signaling in liver injury and offers additional potential targets for drug development in liver diseases. To this end, a lot has yet to be further defined. In this study we revealed transcriptional regulation of SOD2 by PPARγ, which plays central role in the mechanism of DDB mediated hepatoprotective effect. Although our findings demonstrated that DDB attenuates CCl_4_-induced necroptosis via the Nrf2/PPARγ/SOD2 pathway, the present study primarily focused on hepatocytes and did not investigate the potential effects of DDB on necroptosis in non-parenchymal liver cells, such as Kupffer cells or hepatic stellate cells. Second, although the CCl_4_-induced liver injury model is well established and widely used, it reflects acute hepatotoxicity rather than chronic liver disease. Therefore, the translational potential of DDB in conditions such as fibrosis or steatohepatitis warrants further evaluation in models with greater clinical relevance. Nevertheless, we found that DDB regulates PPARγ via acting Nrf2. Importantly, our results show that Nrf2 indirectly regulates SOD2 through the modulation of PPARγ, rather than directly regulating SOD2, thereby reducing mtROS production and inhibiting MLKL activation. This provides new directions and options for exploring new therapies for the prevention and treatment of liver injury.

## Conclusion

This study demonstrated that DDB can effectively alleviate CCl_4_-induced ALI by inhibiting the necroptosis signaling pathway. DDB activated Nrf2-mediated PPARγ transcription, which in turn upregulated SOD2 transcription by binding to the SOD2 promoter, reducing the release of mtROS and inhibiting the activation of MLKL, thereby suppressing the activation of necroptosis and exerting its hepatoprotective effects. These findings indicate that the Nrf2/PPARγ/SOD2 axis contributes to the regulation of mtROS levels and necroptosis activation in liver injury, providing insight into the hepatoprotective mechanism of DDB.

## Supplementary Information

Additional file 1.

## Data Availability

The datasets used and/or analysed during the current study are available from the corresponding author on reasonable request.

## Abbreviations

ALI: Acute liver injury
ALT: Alanine aminotransferase
AREs: Antioxidant response elements
AST: Aspartate aminotransferase
CETSA: Cellular thermal shift assay
ChIP: Chromatin immunoprecipitation
DARTS: Drug affinity responsive target stability
DDB: Dendrobine
Keap1: Kelch-like ECH-associated protein 1
mtROS: Mitochondrial reactive oxygen species
NAFLD: Nonalcoholic fatty liver disease
Nrf2: Nuclear factor erythroid 2-related factor 2
PIO: Pioglitazone
p-MLKL: Phosphorylated mixed lineage kinase-like protein
PPARγ: Peroxisome proliferator-activated receptor gamma
RT: Room temperature
RT-qPCR: Quantitative real-time PCR
SOD2: Superoxide dismutase 2
