# Supplementary material for: Dendrobine alleviates CCl4-induced acute liver injury by inhibiting necroptosis through activation of the Nrf2/PPARγ/SOD2 signaling pathways
Source: Chin Med. 2025 Nov 2;20:182. doi: 10.1186/s13020-025-01241-8 (PMC12579814; doi:10.1186/s13020-025-01241-8)
Supplement: Supplementary file 1 — Additional file 1. [file 13020_2025_1241_MOESM1_ESM.docx]

**Supplementary material**

**Dendrobine alleviates CCl_4_-induced acute liver injury by inhibiting necroptosis through activation of** **the Nrf2/PPARγ/SOD2 signaling pathways**

**Yonggang Yang^a,b^, Xiaolong Fu^a,b^, Ling Tan ^a,b^, Siting Xian^a,b^, Naiyu Fan^a,b^, Qinglin Gan^a,b^, Nan Nan^a,b^,** **Lizhen Hu^a,b^, Jingshan Shi^a,b^, Qin Wu^a,b^, Shaoyu Zhou^a,b,*^**

^a^ Key Laboratory of Basic Pharmacology of Ministry of Education and Joint

International Research Laboratory of Ethnomedicine of Ministry of Education, Zunyi Medical University, Zunyi, China

^b^ School of Pharmacy, Zunyi Medical University, Zunyi, China

*** Correspondence:**

Shaoyu Zhou, Ph.D.

Professor of Pharmacology

Key Laboratory of Basic Pharmacology of Ministry of Education

Zunyi Medical University

Zunyi, Guizhou, China

Email: szhou@zmu.edu.cn

**Supplementary Figures**

**Supplementary Figure 1**


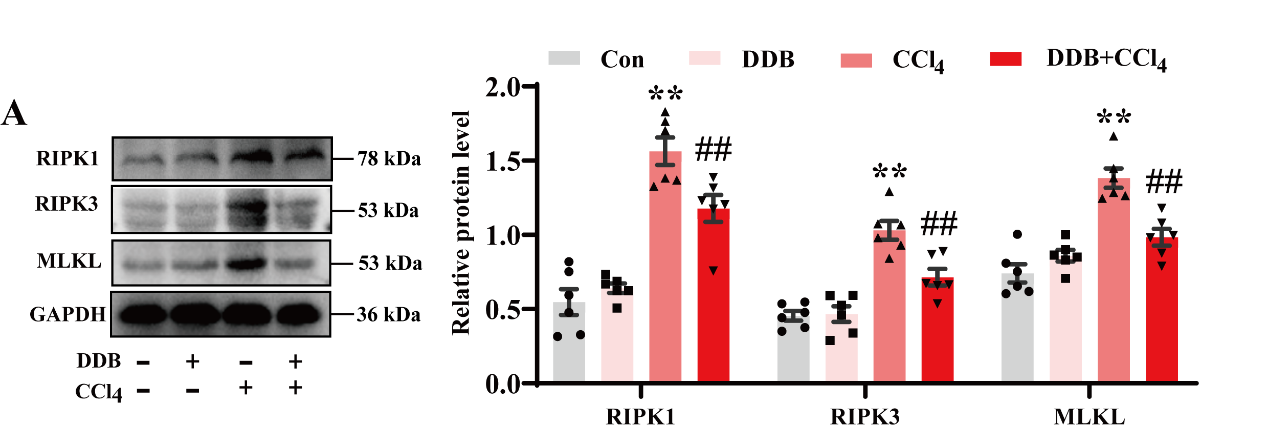


**Fig. S1** DDB alleviates CCl_4_-induced ALI in mice via modulation of necroptotic signaling. **A** Expression levels and quantitative analysis of p-RIPK1, p-RIPK3 and p-MLKL proteins in liver tissues (n = 6). Compared with the control group, **P* < 0.05, ***P* < 0.01; compared with the CCl_4_ group, ^#^*P* < 0.05, ^##^*P* < 0.01.

**Supplementary Figure 2**


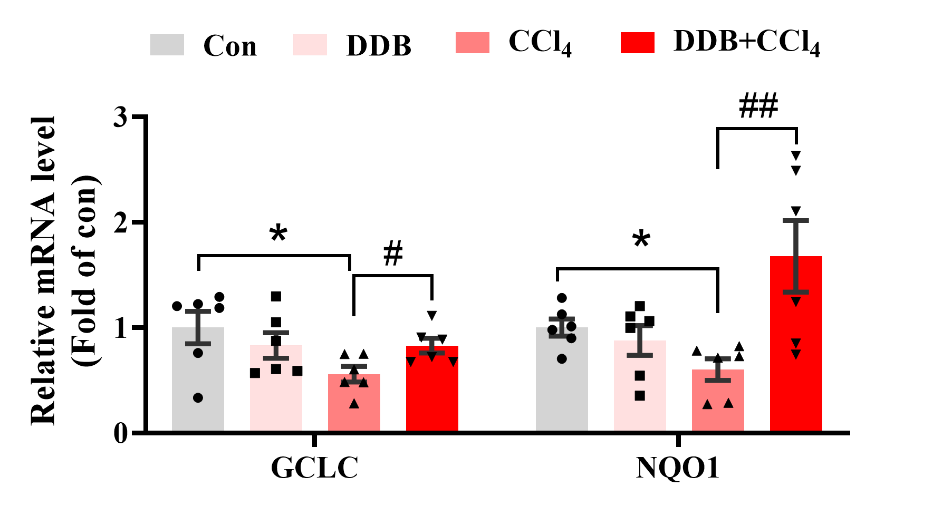


**Fig. S2** DDB affects expression levels of GCLC and NQO1. **A** mRNA expression of GCLC and NQO1 in tissue samples (n = 6). Compared with the control group, **P* < 0.05; compared with the CCl_4_ group, ^#^*P* < 0.05, ^##^*P* < 0.01.


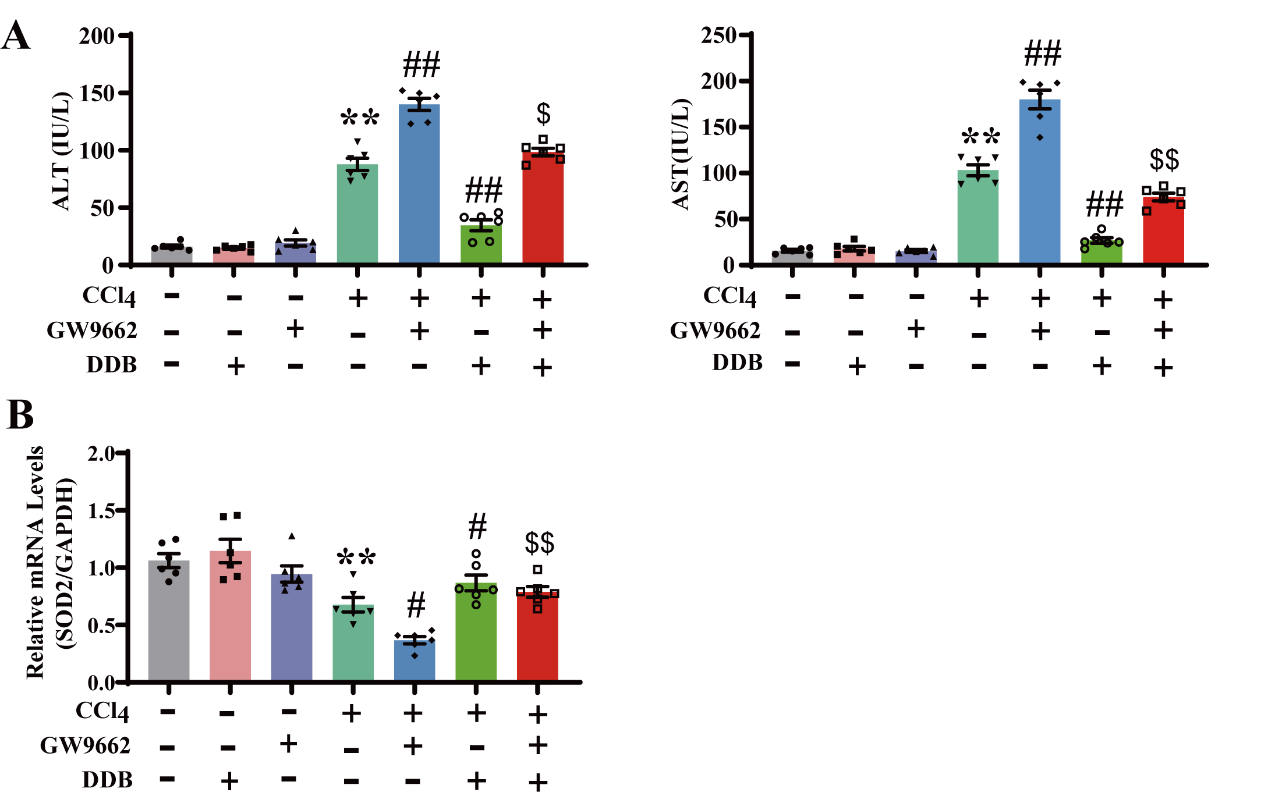
**Supplementary Figure 3**

**Fig. S3** *In vivo* inhibition of PPARγ exacerbates CCl_4_-induced ALI via necroptosis. **A** Serum levels of ALT and AST in mice administered GW9662 *in vivo* (n = 6). **B** mRNA expression of SOD2 in liver tissues (n = 6). Compared with the control group, **P* < 0.05, ***P* < 0.01; compared with the CCl_4_ group, ^#^*P* < 0.05, ^##^*P* < 0.01; compared with the GW9662 + CCl_4_ group, ^$^*P* < 0.05, ^$$^*P* < 0.01.


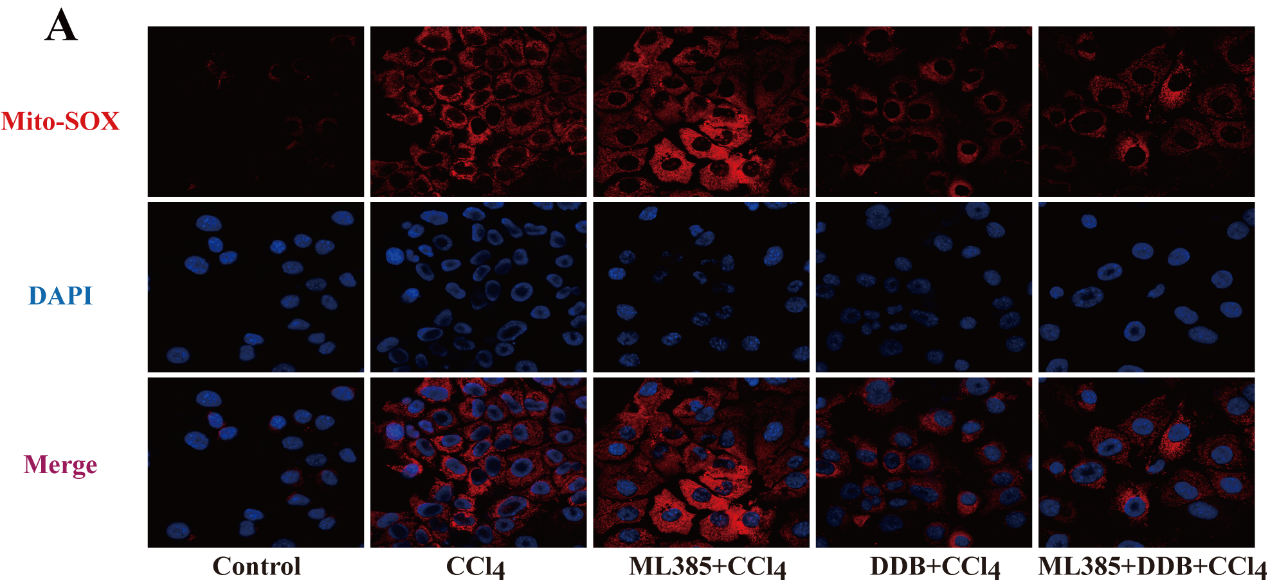
**Supplementary Figure 4**

**Fig. S4** DDB regulates PPARγ and SOD2 expression via Nrf2 to suppress necroptosis. **A** Effects of Nrf2 on mtROS levels in AML-12 cells (n = 3).

**Supplementary Figure 5**


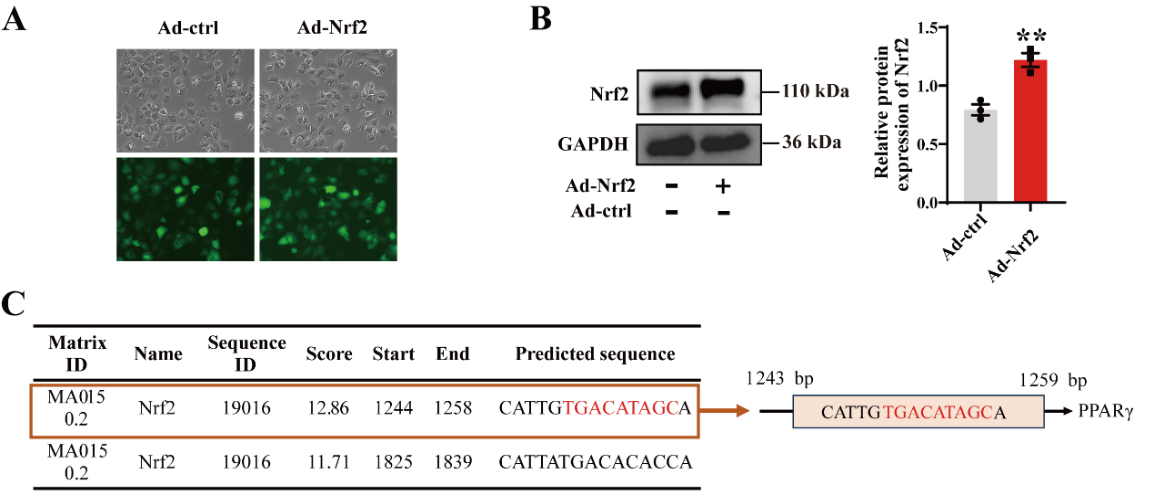


**Fig. S5** Nrf2 directly regulates the expression of PPARγ. **A** Representative fluorescence images of Nrf2 overexpressing cells (n = 3). **B** Representative WB images and quantification of Nrf2 protein in Nrf2 overexpressing cells (n = 3). **C** Predicted binding sites on the promoters of Nrf2 and PPARγ, with red letters indicating ARE. Compared with the control group, ***P* < 0.01.
